# Elevated remnant cholesterol as a potential predictor for cardiovascular events in rheumatoid arthritis patients

**DOI:** 10.3389/fcvm.2024.1449219

**Published:** 2024-09-09

**Authors:** Ching-Kun Chang, Yi-Chen Li, Po-Ku Chen, Shih-Hsin Chang, Der-Yuan Chen

**Affiliations:** ^1^ Rheumatology and Immunology Center, China Medical University Hospital, Taichung, Taiwan; ^2^ College of Medicine, China Medical University, Taichung, Taiwan; ^3^ Translational Medicine Laboratory, Rheumatology and Immunology Center, China Medical University Hospital, Taichung, Taiwan; ^4^ Organ-on-a Chip Fabrication and Verification Division, Taiwan Instrument Research Institute, National Applied Research Laboratories, Hsinchu, Taiwan; ^5^ Clinical Medicine Research Center, National Cheng Kung University Hospital, Tainan, Taiwan; ^6^ Center of Cell Therapy, National Cheng Kung University Hospital, Tainan, Taiwan; ^7^ Institute of Clinical Medicine, College of Medicine, National Cheng Kung University, Tainan, Taiwan; ^8^ Translational Medicine and Rong Hsing Research Center for Translational Medicine, National Chung Hsing University, Taichung, Taiwan; ^9^ Institute of Medicine, Chung Shan Medical University, Taichung, Taiwan

**Keywords:** remnant cholesterol (RC), low-density lipoprotein (LDL), prediction, cardiovascular events (CVE), rheumatoid arthritis (RA)

## Abstract

**Objective:**

The risk of cardiovascular disease (CVD) in patients with rheumatoid arthritis (RA) remains inadequately defined. Consequently, this study aims to evaluate the predictive value of remnant cholesterol (RC) for assessing CVD risk in RA patients.

**Methods:**

Plasma RC levels were measured in 114 RA patients and 41 healthy controls, calculated as total cholesterol minus HDL-C and LDL-C. These levels were further analyzed using ^1^H-NMR lipid/metabolomics. Meanwhile, the 28-joint Disease Activity Score (DAS28) assessed RA activity.

**Results:**

RC levels were significantly elevated in RA patients (19.0 mg/dl, *p* < 0.001) compared to healthy controls (14.5 mg/dl). Furthermore, RC levels were significantly elevated at 37.4 mg/dl in patients who experienced cardiovascular event (CVE) compared to 17.4 mg/dl in those without CVE (*p* < 0.001). To enhance the precision and reliability of RC measurements, RC concentrations were further validated using ^1^H-NMR spectroscopy. Additionally, a positive correlation was observed between RC levels and DAS28. Multivariate analysis identified RC as a significant predictor of CVE (odds ratio = 1.82, *p* = 0.013). ROC curve analysis revealed superior predictive capability of RC for CVE (AUC = 0.919, *p* < 0.001) compared to LDL-C (AUC = 0.669, *p* = 0.018), with a high sensitivity of 94.7% and a specificity of 82.1%.

**Conclusion:**

Elevated RC levels demonstrate greater accuracy in predicting CVE occurrence in RA patients compared to traditional measures such as LDL-C. These findings suggest that elevated RC levels may serve as a novel predictor for occurrence of CVE in RA patients, facilitating early intervention strategies based on the risk stratification.

## 1 Introduction

Atherosclerosis, a chronic inflammatory process, is characterized by atheromatous plaque buildup and is the major cause of cardiovascular disease (CVD) (1). With dyslipidemia being strongly associated with CVD, low-density lipoprotein cholesterol (LDL-C) is conventionally regarded as the most important contributing factor to CVD development (2). Thus, the current treatment guidelines for CVD prevention focus on the reduction of LDL-C levels (3, 4); however, several studies revealed persistent residual CVD risk after optimal statin therapy (5–7). Nordestgaard et al. demonstrated that elevated levels of triglycerides conferred an increased risk of CVD (8). Triglycerides are major components of triglyceride-rich lipoproteins (TGRLs), which include chylomicrons, very low-density lipoproteins (VLDLs), and intermediate-density lipoproteins (IDLs), and are rapidly catabolized by lipoprotein lipase with the production of respective remnants. Remnant cholesterol (RC), the cholesterol carried in TGRLs, is known for its critical pathogenic role in atherosclerosis and CVD (9–15). Like LDL-C, RC can easily enter the arterial wall and get retained in the subendothelial layer, where they are taken up by macrophages, leading to foam cell formation and cholesterol buildup in arterial plaques (15, 16). Compared with LDL-C, RC tends to be more readily taken up by scavenger receptors, thus further facilitating arterial plaque formation (17, 18). High levels of RC can also enhance the expression of inflammatory cytokines and promote vascular inflammation, which is not observed in the presence of high levels of LDL-C (19, 20).

Rheumatoid arthritis (RA), a chronic autoimmune inflammatory disease (21), is complicated by a high CVD burden (22), which is probably due to traditional risk factors, dyslipidemia, and disease-related inflammation (23, 24). We previously revealed an inverse correlation between RA-related inflammation and LDL-C levels (25). Lower LDL-C levels in conjunction with higher CVD risk in RA patients than in the general population support the hypothesis of a lipid paradox in RA (22, 26). This paradox suggests that other forms of atherogenic dyslipidemia, such as RC, might account for the elevated CVD risk in RA patients with low LDL-C levels. LDL-C measurement is now widely implemented in clinical practice to evaluate CVD risk in RA patients. If circulating RC levels could predict CVD risk or events in RA patients, which has yet to be explored, they might serve as an important CVD risk predictor in this disease. Since RC is calculated as TC−LDL−HDL, and bDMARDs and JAK inhibitors have been shown to significantly affect LDL and HDL levels (27, 28), it is plausible that these treatments may also influence RC levels. Exploring the impact of these medications on RC is a key focus of our study.

With a link between elevated RC levels and inflammation as well as the emergence of CVD (9–14), we speculated that RA patients would exhibit elevated RC levels. Given a lipid paradox in RA (20, 24), cardiovascular event (CVE) risks may not parallel LDL-C levels in RA patients. Besides, the relationship between RC levels and CVE in RA patients has scarcely been explored. Hence, this cross-sectional and prospective study aimed to compare the plasma RC levels between RA patients and healthy control (HC) participants and between patients with and without CVE. The utility of plasma RC levels for predicting incident CVE was evaluated in patients with RA during longitudinal follow-up. With RA being an inflammatory disease, we expect a positive correlation between RC levels and RA disease activity. Given that RC is the cholesterol carried in triglyceride-rich lipoproteins (TGRLs), we expect a positive correlation between RC and TG levels. Therefore, we also examined the correlation between plasma RC levels and plasma levels of other lipid profiles or RA disease activity. Accumulative evidence indicates the significant influence of biologics and JAK inhibitors on levels of lipid profile in RA patients. We finally investigated the effects of biologic disease-modifying anti-rheumatic drugs (bDMARDs) or Janus kinase inhibitors (JAKi) on plasma RC levels in RA patients.

## 2 Methods and materials

### 2.1 Sample

This prospective, single-centered, and cross-sectional study consecutively enrolled 114 patients who fulfilled the 2010 classification criteria of the American College of Rheumatology/European League Against Rheumatism (ACR/EULAR) collaborative initiative for RA (29). Eligible criteria for inclusion were as follows: [1] age at study entry older than 20 years, [2] a follow-up period longer than 12 months after investigation for lipid profile and lipid metabolites, and [3] active disease with a poor therapeutic response to the conventional synthetic DMARDs (csDMARDs). Patients were excluded if they were in the acute phase of myocardial infarction (AMI) or had an ischemic stroke within three months before enrollment in the present study. Disease activity was assessed using DAS28-erythrocyte sedimentation rate (DAS28-ESR) (30), and active status was defined as DAS28 ≥ 3.2. After baseline lipid examination, all 114 active RA patients started bDMARDs or JAKi therapy according to the recommendations (31). This study also enrolled forty-one healthy subjects with no history of rheumatic disease or CVD as HC. The China Medical University and Hospital Research Ethics Center approved this study (CMUH109-REC3-161, approval date 13 December 2020), and each participant's written consent was obtained according to the Declaration of Helsinki.

### 2.2 Measurements of lipid profiles, atherogenic index, and RC

All the blood samples were collected from the participants in the early morning after an overnight fast for at least 10 h. The blood samples were then used to determine the plasma levels of total cholesterol, triglycerides, high-density lipoprotein cholesterol (HDL-C), and LDL-C. These examinations were accurately carried out utilizing enzymatic methodologies on a Beckman Coulter AU5800 chemistry analyzer (California, USA), strictly following the manufacturer's instructions. The atherogenic index was derived by precisely calculating the ratio of total cholesterol to HDL-C. Plasma RC levels were calculated as the levels of total cholesterol minus HDL-C minus the measured LDL-C (32, 33).

### 2.3 Determination of circulating RC levels by ^1^H-NMR lipid/metabolomics

Circulating levels of RC were analyzed using the ^1^H-NMR lipid/metabolomics (*N*ightingale Health, Helsinki, Finland) as in previous studies (34, 35). In detail, 100*μ*l serum and phosphate buffer (prepared with 5.5 mM sodium 3-trimethylsilyl [2,2,3,3-d4] propionate, 0.075 M Na_2_HPO_4_*7H_2_O, 5 ml NaN_3_ (4%) adjusted to pH 7.4 with 1 M HCl) were mixed in an Eppendorf tube. The samples were transferred to a 3 mm NMR tube (Bruker Match system) and measured at 310 K in Bruker Avance III NMR spectrometers operating at 600.13 MHz equipped with a maximum gradient strength of 53 G/cm. Each data set was automatically processed using a line broadening of 1 Hz, with the NOESY data aligned to the alanine signal at 1.49 ppm.

### 2.4 Measurement of CVE outcomes

The endpoint events in the present study were the incident CVE during the follow-up period, including fatal or non-fatal MI, stable or unstable angina pectoris, ischemic stroke, and reversible focal neurological defects with imaging evidence of a new cerebral lesion compatible with ischemia (36).

### 2.5 Statistical analysis

The data were presented as the mean ± standard deviation (SD) or the median (interquartile range, IQR). Given the unequal group sizes and small sample size, we opted for non-parametric statistical methods to account for potential non-normal distributions. We performed a chi-squared test to examine the between-group difference of categorical variables. The Mann-Whitney *U*-test was used for between-group comparison of numerical variables, and the correlation coefficient was obtained through the nonparametric Spearman's rank correlation test. The Wilcoxon matched-pairs signed-rank test was used to compare the plasma levels of RC and other lipid profile in RA patients before and after treatment. We constructed a univariate and multiple logistic regression model to evaluate factors contributing to the emergence of CVE, including age, gender, smoking, and lipid profile. The optimal cut-off level of RC for predicting the presence of CVE in RA patients was determined by the receiver operating characteristic (ROC) curve analysis. Mean and SD results were included in Supplementary Tables S1 and S2 to provide a comprehensive view alongside the median and IQR. A two-sided *p*-value < 0.05 was considered statistically significant. The plots and statistical analysis were performed using IBM SPSS Statistics v25 (IBM, New York, USA) and GraphPad Prism v9.3 (GraphPad Software, San Diego, USA).

## 3 Results

### 3.1 Clinical characteristics and lipid profile of RA patients vs. HC

In this study, age and gender were well-matched between RA patients and the HC group (Table 1). There were no significant differences in the proportion of females or body mass index (BMI) between the two groups. RC levels were notably higher in the RA group than in the HC group (19.0 mg/dl vs. 14.5 mg/dl, *p* < 0.001). No significant differences were observed in triglycerides, total cholesterol, LDL-C, HDL-C, or the atherogenic index between the groups.

**Table 1:** Demographic data and laboratory findings in rheumatoid arthritis (RA) patients and healthy control participants^a^.

	RA (*n* = 114)	Healthy control (*n* = 41)
Age at entry, years	60.5 (54.8–65.0)	55.0 (48.0–66.0)
Female proportion, *n* (%)	88 (77.2%)	29 (70.7%)
Body mass index, kg/m^2^	23.3 (20.7–26.3)	23.0 (21.2–24.2)
Total cholesterol, mg/dl	193.5 (165.5–223.3)	186.0 (159.5–223.5)
Triglyceride, mg/dl	87.0 (69.5–128.5)	79.0 (64.0–118.8)
LDL-C, mg/dl	110.5 (84.8–137.2)	111.0 (86.2–143.4)
HDL-C, mg/dl	61.2 (49.1–71.3)	61.0 (50.6–75.1)
Atherogenic index	3.16 (2.63–3.89)	3.03 (2.55–3.96)
RC, mg/dl	19.0 (14.3–27.6)	14.5 (7.7–17.7)**
Hypertension, *n* (%)	29 (25.4%)	2 (9.5%)
Diabetes mellitus, *n* (%)	6 (5.3%)	0 (0%)
Current smoker, *n* (%)	16 (14.0%)	3 (14.3%)

^a^ Data are presented as median (interquartile range, IQR) or number (percentage). *P*-values assessing between-group differences were derived from the chi-squared test for categorical variables and the Mann-Whitney *U*-test for numerical variables. Statistically significant differences are indicated in bold, with * denoting *p* < 0.05 and ** denoting *p* < 0.01. RC, remnant cholesterol.

### 3.2 Clinical characteristics and lipid profile of RA patients with and without incident CVE

During a mean follow-up period of 27 months, 19 (16.7%) RA patients developed incident CVE (Table 2). Patients with CVE exhibited significantly higher levels of total cholesterol, triglycerides, LDL-C, atherogenic index, and RC compared to those without CVE. There were no significant differences in gender proportion, BMI, disease duration, rheumatoid factor (RF) or anti-citrullinated peptide antibodies (ACPA) positivity, disease activity scores, inflammatory parameters, or prescribed medications.

**Table 2:** Demographic data and laboratory findings in RA patients with or without cardiovascular event (CVE)^a^.

	RA with CVE (*n* = 19)	RA without CVE (*n* = 95)
Age at entry, years	65.0 (64.0–72.0)	59.0 (54.0–65.0)**
Female proportion, *n* (%)	12 (63.2%)	76 (80.0%)
Disease duration, years	7.0 (5.7–10.5)	6.7 (5.3–8.2)
Body mass index, kg/m^2^	24.3 (22.0–28.1)	23.1 (20.6–26.1)
RF positivity, *n* (%)	12 (63.2%)	70 (73.7%)
ACPA positivity, *n* (%)	12 (63.2%)	72 (76.6%)
Baseline ESR, mm/1st hr	19.0 (16.0–44.0)	25.0 (14.0–44.0)
Baseline CRP, mg/dl	0.74 (0.12–1.20)	0.85 (0.17–2.41)
DAS28-ESR at baseline	6.51 (5.42–6.72)	5.72 (4.98–6.44)
Total cholesterol, mg/dl	227.0 (194.0–268.0)	187.0 (163.0–220.0)**
Triglyceride, mg/dl	158.0 (101.0–197.0)	84.0 (63.5–113.8)**
LDL-C, mg/dl	124.5 (109.2–157.9)	105.4 (84.0–135.4)*
HDL-C, mg/dl	59.6 (47.4–65.4)	61.8 (51.0–72.1)
Atherogenic index	3.99 (3.07–4.82)	3.12 (2.57–3.58)**
RC, mg/dl	37.4 (26.9–44.9)	17.4 (13.7–22.7)**
Daily corticosteroids, mg/day	4.0 (0.0–4.0)	5.0 (2.5–8.0)
Baseline used csDMARDs		
Methotrexate, *n* (%)	16 (84.2%)	76 (80.0%)
Sulfasalazine, *n* (%)	13 (68.4%)	67 (70.5%)
Hydroxychloroquine, *n* (%)	12 (63.2%)	58 (61.1%)
bDMARDs/JAKi after lipid investigation		
TNF-α inhibitors, *n* (%)	3 (15.8%)	18 (18.9%)
Non-TNF-α inhibitors, *n* (%)	7 (36.8%)	29 (30.5%)
JAK inhibitors, *n* (%)	9 (47.4%)	48 (50.5%)
The use of statin, *n* (%)	10 (52.6%)	20 (21.1%)**
Hypertension, *n* (%)	9 (47.4%)	20 (21.1%)*
Diabetes mellitus, *n* (%)	2 (10.5%)	4 (4.2%)
Current smoker, *n* (%)	1 (5.3%)	15 (15.8%)

^a^ Data are presented as median (interquartile range, IQR) or number (percentage). RA, rheumatoid arthritis; RF, rheumatoid factor; ACPA, anti-citrullinated peptide antibodies; ESR, erythrocyte sedimentation rate; CRP, C-reactive protein; DAS28, disease activity score for 28-joints; LDL, low-density lipoprotein; HDL, high-density lipoprotein; RC, remnant cholesterol; csDMARDs, conventional synthetic disease-modifying anti-rheumatic drugs; bDMARDs, biologic DMARDs; JAKi, Janus kinase inhibitors; TNF, tumor necrosis factor. *P*-values assessing between-group differences were derived from the chi-squared test for categorical variables and the Mann-Whitney *U*-test for numerical variables. Statistically significant differences are indicated in bold, with * denoting *p* < 0.05 and ** denoting *p* < 0.01.

### 3.3 Comparisons of plasma levels of RC and lipid profile between RA patients and HC participants, or between RA patients with and without incident CVE

**Figure 1:**
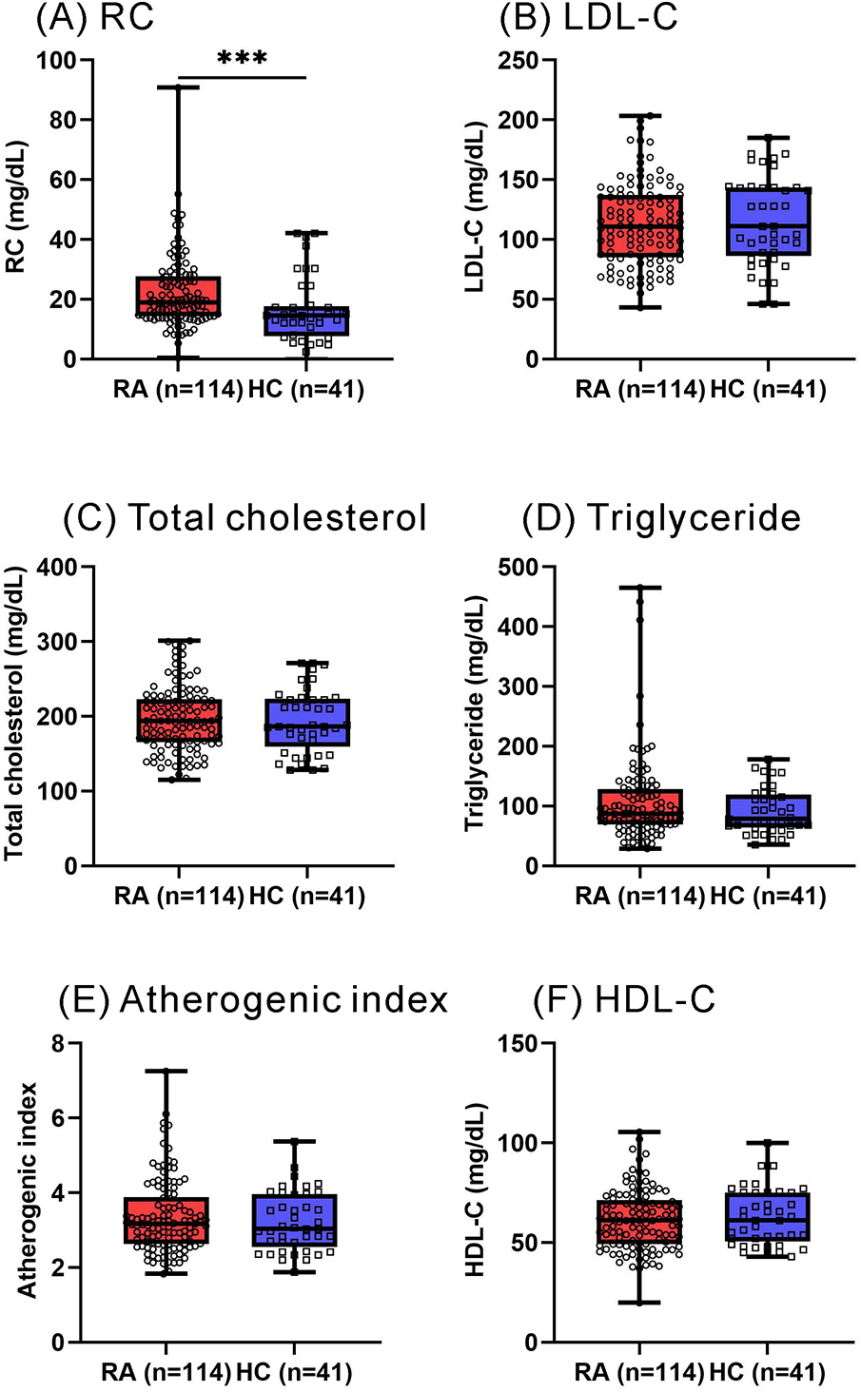
Plasma levels of RC and lipid profile between RA patients and HC participants. **(A)** RC **(B)** LDL-C **(C)** total cholesterol **(D)** triglyceride **(E)** atherogenic index **(F)** HDL-C. RA: rheumatoid arthritis; RC: remnant cholesterol; LDL-C: low-density lipoprotein cholesterol; HDL-C: high-density lipoprotein cholesterol. *p < 0.05, **p < 0.005, ***p < 0.001, determined by using Mann–Whitney *U*-test.

**Figure 2:**
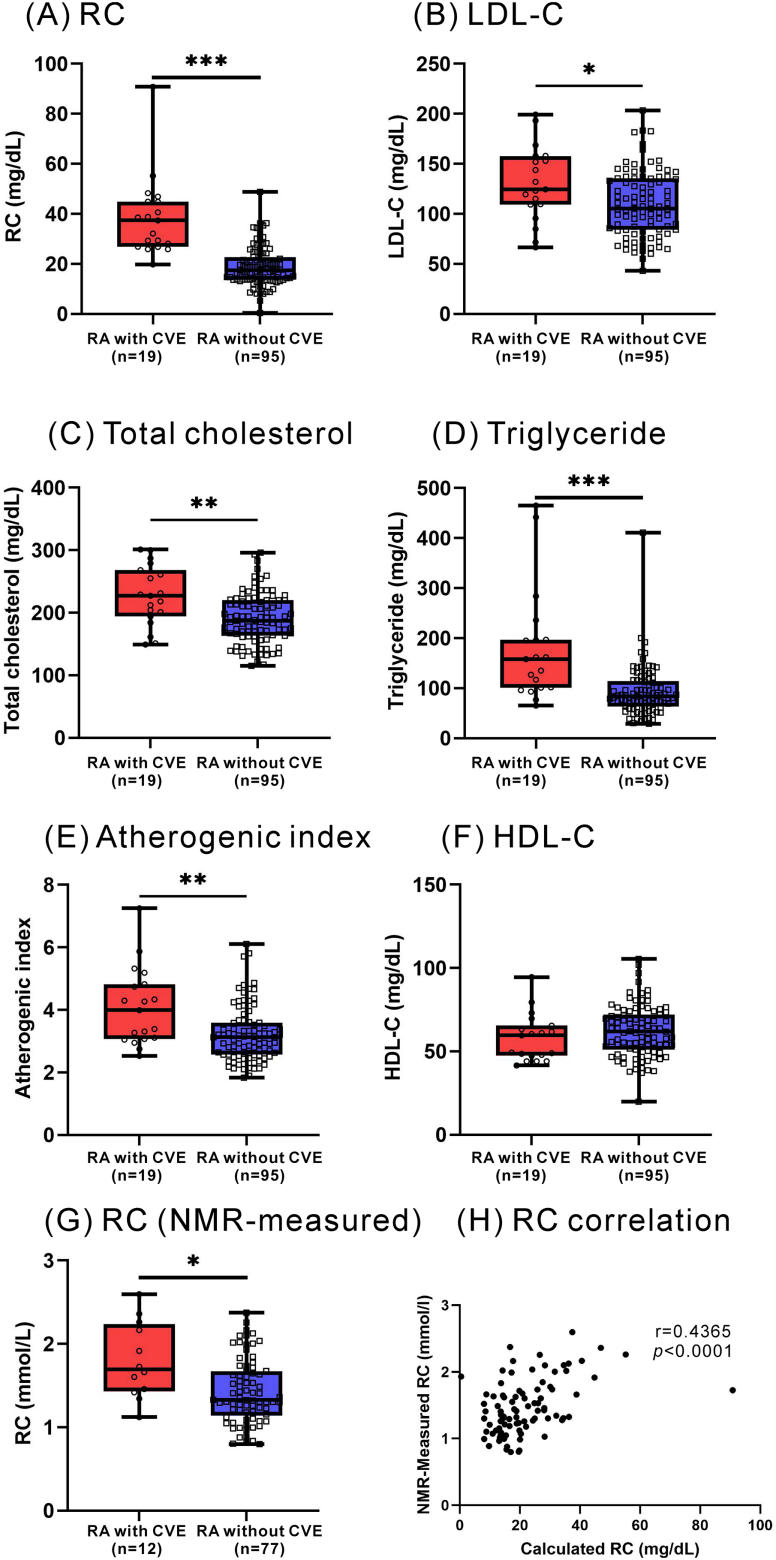
Plasma levels of RC and lipid profile between RA with and without incident CVE. **(A)** RC **(B)** LDL-C **(C)** total cholesterol **(D)** triglyceride **(E)** atherogenic index **(F)** HDL-C **(G)** NMR-measured RC. **(H)** The correlation between NMR-measured RC and calculated RC. *p < 0.05, **p < 0.005, ***p < 0.001, determined by using Mann–Whitney *U*-test.

As illustrated in Figure 1, RA patients had significantly higher calculated RC levels at baseline (median 19.0 mg/dl, IQR 14.3–27.6 mg/dl) compared with healthy participants (14.5 mg/dl, IQR 7.7–17.7 mg/dl, *p *< 0.001). However, there were no significant differences in plasma levels of lipid profile or atherogenic index between RA patients and HC participants. As shown in Figure 2, significantly higher calculated RC levels at baseline were observed in patients with CVE than in those without CVE (37.4 mg/dl vs. 17.4 mg/dl, *p *< 0.001). RA patients with CVE also had significantly higher levels of total cholesterol, triglycerides, LDL-C, and atherogenic index (median, 227.0 mg/dl, 158.0 mg/dl, 124.5 mg/dl, and 3.99, respectively) than those without CVE (187.0 mg/dl, *p *< 0.005; 84.0 mg/dl, *p* < 0.001; 105.4 mg/dl, *p* < 0.05; and 3.12, *p* < 0.005; respectively). However, there was no significant difference in plasma HDL-C levels between RA patients with and without CVE. To verify the accuracy of RC measurements, we selected nearly 80% of the samples from our initial cohort for additional NMR analysis. As illustrated in Figure 2G, significant differences in RC levels were confirmed, even with this high-precision technique. This finding underscores the clinical importance of RC and affirms the reliability and accuracy of our measurement approach.

### 3.4 Correlation between calculated RC levels and lipid profile, atherogenic index, and disease activity scores (DAS28-ESR) in RA patients

**Figure 3:**
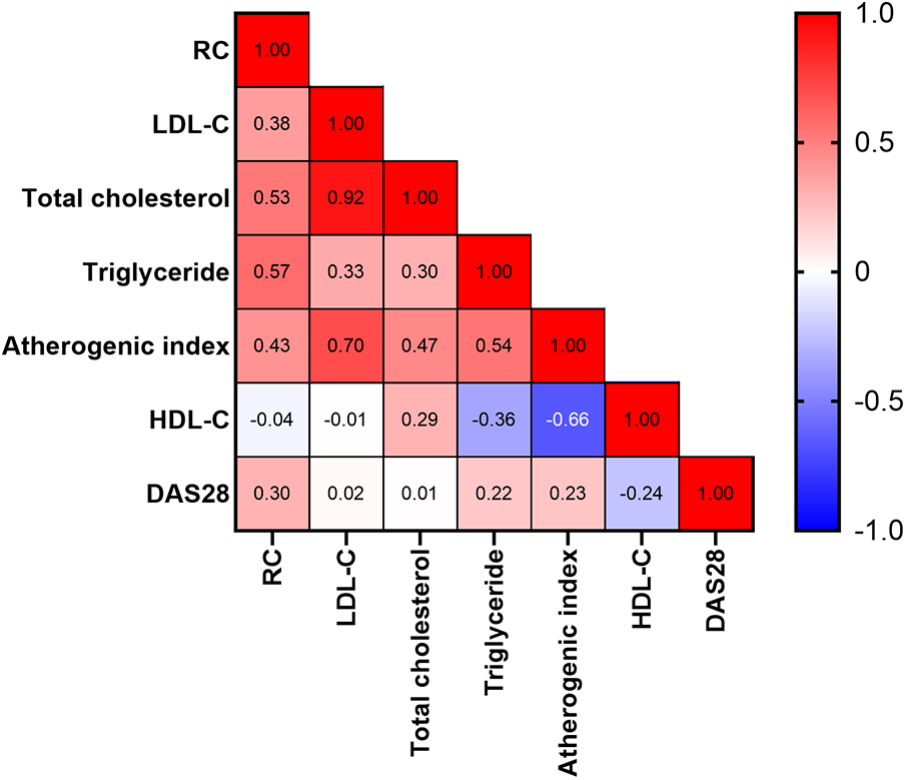
Correlation between calculated RC levels and lipid profile or RA activity scores. The numbers in the squares represent correlation coefficients. DAS28: The 28-joint disease activity score.

As illustrated in Figure 3, the calculated RC levels were positively correlated with plasma levels of triglycerides, total cholesterol, and LDL-C, atherogenic index, and DAS28-ESR scores, respectively, in RA patients. However, there was no significant correlation between calculated RC levels and HDL-C levels in RA patients.

**Figure 4:**
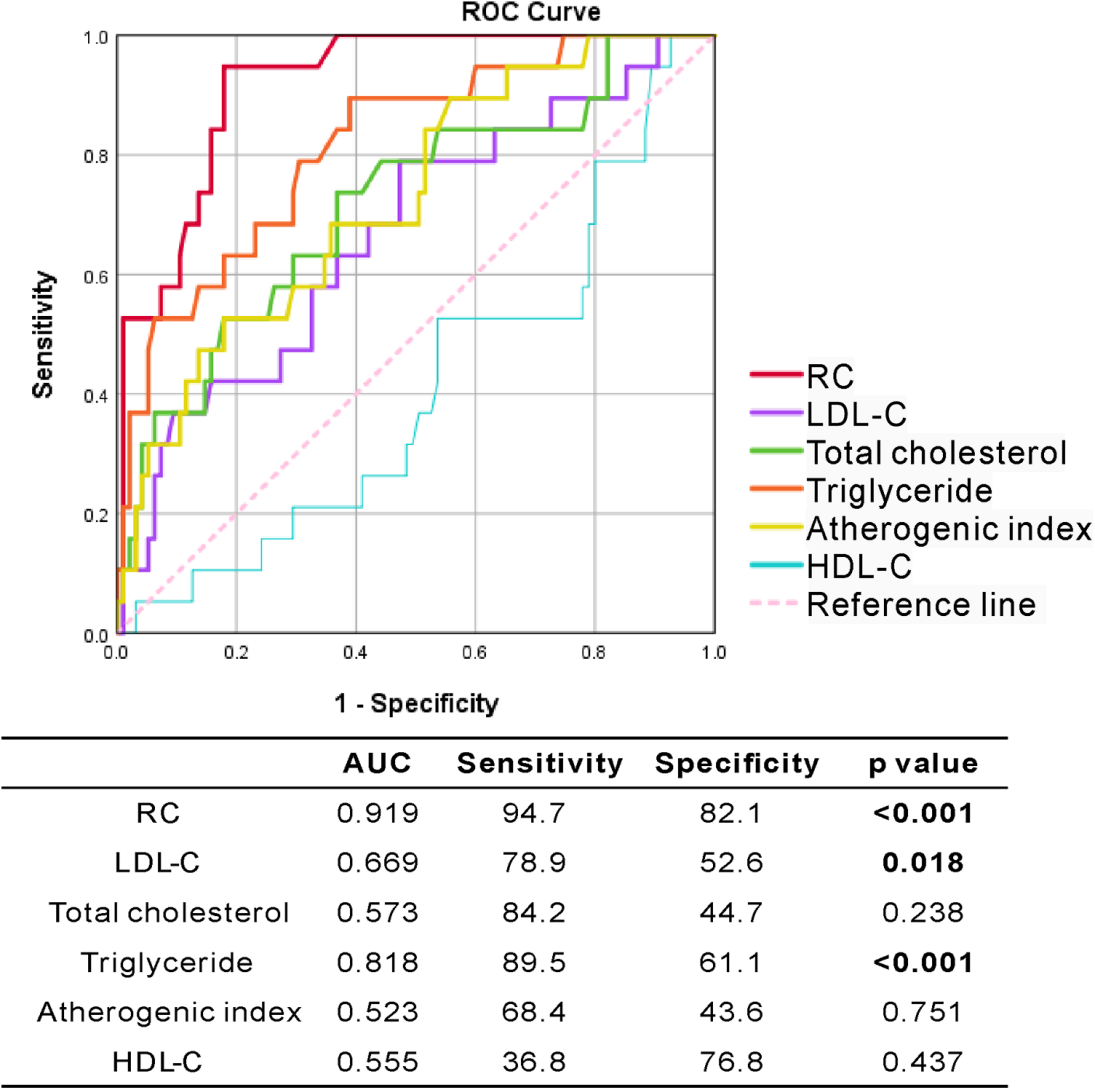
The ROC curve analysis of RC and lipid profile for predicting incident CVE in RA patients.

### 3.5 Logistic regression and ROC analysis for predicting the incident CVE

To establish the best model to predict the emergence of CVE, a multivariate logistic regression analysis was performed. As illustrated in Table 3, the univariate regression analysis identified age, LDL-C, total cholesterol, triglycerides, atherogenic index, and RC levels as the potential predictors of the emergence of CVE. The multivariate regression analysis demonstrated that age and RC levels were the significant predictors of incident CVE in RA patients. To address potential confounding factors, particularly the effects of different treatment types, we included the three treatment types [tumor necrosis factor (TNF)-*α* inhibitors, non-TNF inhibitors, and JAKi] as covariates in the regression model, as shown in Supplementary Table S3.

**Table 3:** Univariate and multivariate regression analysis of baseline lipid profile and RC for predicting the incident cardiovascular events in 114 patients with RA.

Risk factors (**univariate**)	Odds ratio	95% confidence interval	*p*-value
**Age**	**1**.**077**	**1.022**–**1.135**	**0**.**006**
Gender (Female)	0.429	0.149–1.236	0.117
**Total cholesterol**	**1**.**020**	**1.008**–**1.033**	**0**.**002**
**Triglyceride**	**1**.**017**	**1.007**–**1.027**	**0**.**001**
**LDL-C**	**1**.**018**	**1.003**–**1.033**	**0**.**018**
HDL-C	0.982	0.949–1.016	0.299
**Atherogenic index**	**2**.**225**	**1.361**–**3.636**	**0**.**001**
**ESR**	**0**.**996**	**0.971**–**1.021**	**0**.**740**
**CRP**	**0**.**959**	**0.807**–**1.140**	**0**.**634**
**DAS28**	**1**.**526**	**0.941**–**2.472**	**0**.**086**
**RC**	**1**.**198**	**1.110**–**1.293**	**<0**.**001**
Risk factors (**multivariate**)	Odds ratio	95% confidence interval	***p*-value**
**Age**	**1**.**274**	**1.066**–**1.523**	**0**.**008**
Gender (Female)	0.111	0.009–1.347	0.084
Total cholesterol	0.851	0.657–1.102	0.220
Triglyceride	1.003	0.979–1.028	0.805
LDL-C	1.220	0.858–1.735	0.268
Atherogenic index	0.054	0.000–30.999	0.368
ESR	0.964	0.892–1.042	0.360
CRP	1.060	0.845–1.593	0.358
DAS28	1.204	0.418–3.467	0.731
**RC**	**1**.**847**	**1.060**–**3.219**	**0**.**030**

The incident cardiovascular events include fatal or non-fatal MI, stable or unstable angina pectoris, ischemic stroke, and reversible focal neurological defects with imaging evidence of a new cerebral lesion compatible with ischemia.

RA, rheumatoid arthritis; HDL-C, high-density lipoprotein cholesterol; LDL-C, low-density lipoprotein cholesterol; RC, remnant cholesterol; Atherogenic index corresponds to the ratio of total cholesterol/HDL-C.

Bold values indicate statistically significant results.

Using ROC curve analysis, we evaluated the performance of RC, lipid profile, and atherogenic index in predicting the incident CVE in RA patients. As shown in Figure 4, the performance of RC was better at predicting the occurrence of CVE (AUC 0.919) than that of triglycerides (AUC 0.818) and LDL-C (AUC 0.669). Calculated RC at the cut-off level of 25.8 mg/dl showed the highest predictive power, with a sensitivity of 94.7%, specificity of 82.1%, and accuracy of 91.9% (*p* < 0.001).

### 3.6 Changes of plasma levels of RC and lipid profile in RA patients treated with bDMARDs or JAKi

**Figure 5:**
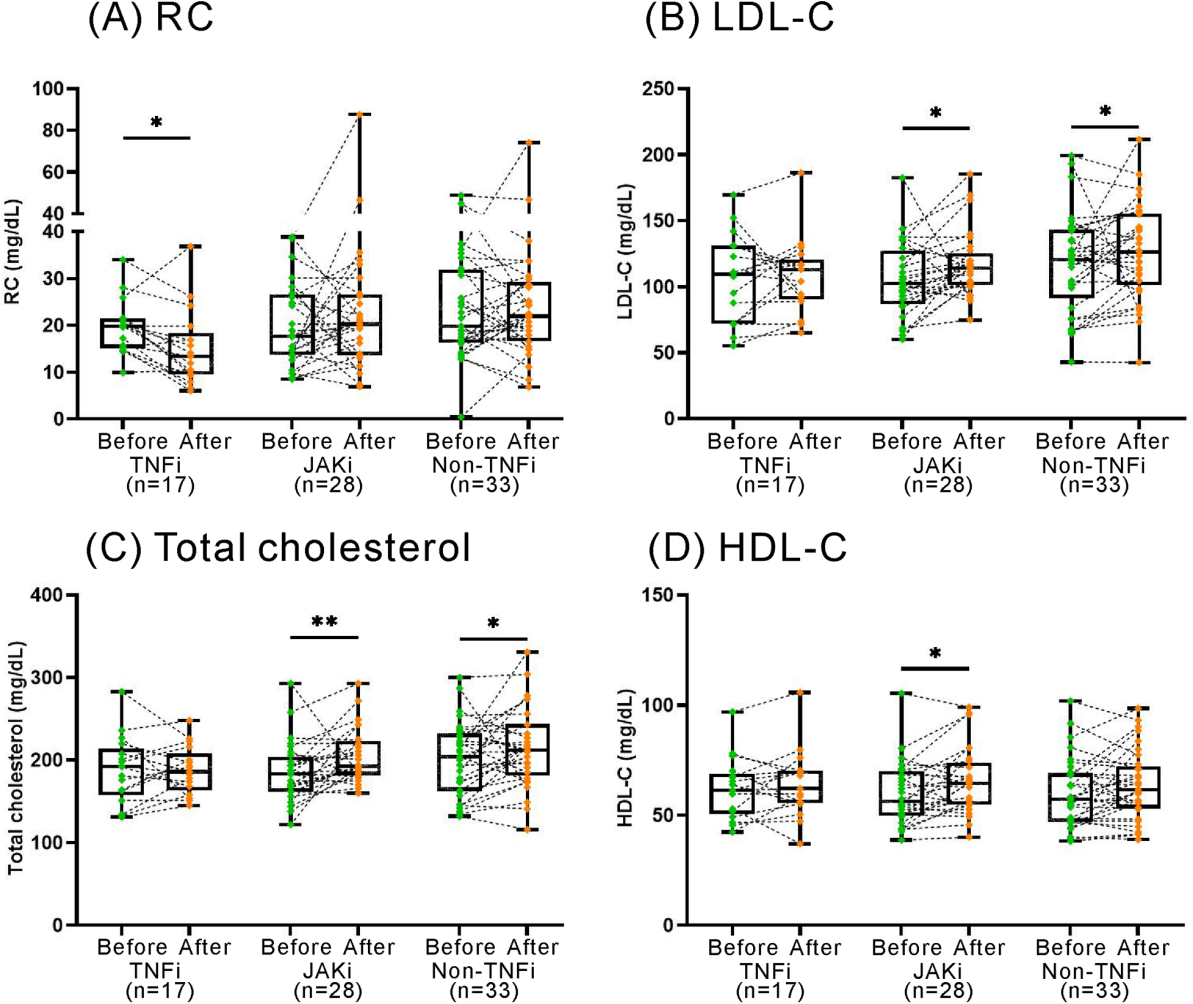
The changes in RC and lipid profile after the therapies with TNFi, non-TNFi, JAKi. TNFi: tumor necrosis factor inhibitors; JAKi: Janus kinase inhibitors. The results are represented by box-plots, where the upper edge of the box indicates the 25th percentile, and the lower edge signifies the 75th percentile. The individual data points are shown as connected dots. *p* < 0.05, determined by using Wilcoxon matched-pairs signed rank test.

Seventy-eight patients were available for evaluation of lipid profile before and after 12 months of treatment with TNF-α inhibitors (TNFi, *n* = 17), non-TNF inhibitors (*n* = 33), or JAKi (*n* = 28). Figure 5 illustrates that TNFi treatment resulted in a significant reduction of plasma RC levels (median 19.8 mg/dl, IQR 15.1–21.5 mg/dl vs. 13.4 mg/dl, IQR 9.5–18.4 mg/dl, *p *< 0.05). In contrast, no significant changes in RC levels were observed in non-TNF inhibitors or JAKi treatment groups. Regarding the changes in other lipid profile, treatment with non-TNFi or JAKi resulted in a significant elevation of total cholesterol levels (median 204 mg/dl, IQR 163–232 mg/dl vs. 212 mg/dl, IQR 182–244 mg/dl; 184 mg/dl, IQR 161–204 mg/dl vs. 193 mg/dl, IQR 182–223 mg/dl, respectively, both *p *< 0.05) and LDL-C levels (121 mg/dl, IQR 92–143 mg/dl vs. 126 mg/dl, IQR 101–156 mg/dl, *p *< 0.05; 103 mg/dl, IQR 87–128 mg/dl vs. 114 mg/dl, IQR 101–126 mg/dl, *p *< 0.005; respectively). However, there were no significant changes in plasma levels of total cholesterol, LDL-C, or HDL-C in TNFi-treated patients.

## 4 Discussion

With the strong association between elevated RC levels and CVD risk (9–15), the predictive value of RC levels for CVD in RA patients is worth investigating. Herein, we demonstrated that plasma RC levels were significantly higher in RA patients than in healthy participants and further higher in patients with CVE than in those without CVE. The calculated RC levels were also positively correlated with RA disease activity reflected by DAS28-ESR scores, triglycerides, total cholesterol, LDL-C, and atherogenic index in RA patients. The multivariate logistic regression analysis further demonstrated RC as a significant predictor of CVE. The ability of RC to predict CVE was better than that of triglycerides or LDL-C, and the ROC curve analysis illustrated that RC threshold at 25.8 mg/dl could predict incident CVE with a high sensitivity of 94.7%, specificity of 82.1%, and accuracy of 91.9%. Besides, the decrease in plasma RC levels paralleled the improvement in disease activity scores in RA patients treated with TNFi. These observations suggest that RC is a useful predictor of incident CVE in RA patients.

Although current guidelines recommend reducing LDL-C levels to curtail CVD risk (3, 4), there is residual CVD risk in some patients despite achieving low LDL-C levels (5–7). Cumulative evidence indicates that TGRLs and triglycerides may contribute to residual CVD risk (8, 14, 15). RC, the cholesterol component of TGRLs such as chylomicron and VLDL/IDL remnants (37), is considered a causal risk factor for CVE (14). Plasma RC level are primarily assessed using two methods: calculated and direct measurement (9, 32, 33). Due to the difficulty in separating RC from other lipoproteins, its calculation involves subtracting HDL-C and LDL-C from total cholesterol (32, 33). This study also employed NMR to validate the reliability of the calculation method, finding consistency between the two approaches. This consistency enhances the credibility and internal coherence of the measurements, demonstrating the reliability of RC quantification through different technological methods. The calculated RC concentrations can inform clinical practice about the risk of CVD. Furthermore, as plasma triglyceride and RC are components of TGRL, they are highly correlated in our assessments of cardiovascular risk.

In RA patients, the elevated insulin resistance (38) would enhance hepatic VLDL production and reduce TGRLs clearance. Accordingly, our RA patients had significantly higher plasma RC levels than healthy participants. Yan et al. utilized the National Health and Nutrition Examination Survey (NHANES) database involving 7,777 eligible participants and also identified a positive correlation between RC and RA (39). Besides, the elevated levels of RC may partly contribute to the high CVD burden in RA patients (22). Huh et al. likewise revealed a significant association of elevated RC with increased CVD risk in patients with type 2 diabetes mellitus (40), in which the CVD risk was equivalent to that in RA (41). Regarding the causal moiety in the atherogenic lipid profile, RC and LDL-C share the capacity to penetrate arterial intima and promote atherosclerosis development by delivering pathogenic cholesterol and forming foam cells (42). However, increased RC levels, but not LDL-C levels, were shown to be responsible for low-grade inflammation and increased secretion of atherogenic molecules, providing mechanistic evidence for RC as a key factor behind CVD (15, 16). In this study, plasma RC levels were also significantly higher in RA patients with CVE than in those without CVE. Our results were consistent with the findings that circulating RC levels were positively related to incident CVE among Chinese patients with RA independent of known risk factors (43). Besides, the multivariate logistic regression analysis, including lipid profile, RA-related inflammatory markers, and the used medications, revealed RC as a significant predictor for incident CVE in our RA patients, even those achieving optimal LDL-C levels. Although the studied disease is different, an elevated RC level is still a significant predictor for CVE in patients with psoriatic arthritis or psoriasis (44). In our ROC analysis, the prediction for CVE using RC was superior to that using LDL-C in RA patients, and RC levels above 25.8 mg/dl could discriminate RA patients with an increased CVE, with a high sensitivity, specificity, and accuracy. Hence, plasma RC level might be a useful and novel predictor for the incident CVE in RA patients. However, the usefulness of this cut-off level in clinical practice needs further validation.

Besides the traditional risk factors, RA-related inflammation would increase CVD risk in RA patients (23, 24). We revealed a positive correlation between plasma RC levels and RA disease activity, as reflected by DAS28-ESR scores. To pursue a treat-to-target goal of reducing inflammation-related sequels (45), RA patients would be prescribed b/ts DMARDs if they were refractory to csDMARDs treatment (46). Increasing evidence reveals that targeting inflammation with TNFi is associated with a decreased risk of CVD and CVE (47). Similarly, plasma RC levels in our RA patients were significantly decreased after TNFi therapy, paralleling the reduction of disease activity. Recently, Kastrati et al. revealed that non-TNF inhibitors did not differ from csDMARDs regarding the influence on CVE risk (48). Accumulative evidence indicates a significant influence of JAK inhibitors on levels of lipid profile such as LDL-C and HDL-C in RA patients (49, 50). Souto et al. reported increased levels of total cholesterol and LDL-C after therapies with tocilizumab or tofacitinib but not with TNFi (49). Resonated with a meta-analysis that CVD risk remained unchanged in upadacitinib-treated patients (50), no significant changes in plasma RC levels were observed in our JAKi-treated patients. Likewise, plasma RC levels did not show significant changes in our RA patients receiving non-TNF inhibitors therapy, despite an elevation of plasma levels of total cholesterol and LDL-C.

Despite the novel findings, there are still some limitations in this pilot study. All subjects in this study are of Chinese ethnicity, so our findings may not be generalizable to other ethnic groups. The concomitant treatment with statin, corticosteroids, or csDMARDs in RA patients may affect lipid profile (51–53). Additionally, as our study design is cross-sectional, it captures data at a single point in time, which primarily provides valuable insights into correlations and associations. Further longitudinal studies would be beneficial to confirm these observations and assess the long-term predictive value. Given the small sample size of our RA patients with incident CVE, a future large-scale multiethnic study is required to confirm our findings.

## 5 Conclusion

This is the first to demonstrate that plasma RC levels were significantly elevated in RA patients and were even higher in those with incident CVE. A potential link was observed between RC levels and RA disease activity. Plasma RC level above 25.8 mg/dl could predict incident CVE in RA patients, independent of LDL-C levels. Nevertheless, the causative role of RC in RA-related CVE still needs further investigation.

## Data Availability

The original contributions presented in the study are included in the article/Supplementary Material, further inquiries can be directed to the corresponding author.

## References

[1] Libby P. Inflammation in atherosclerosis. Arterioscler Thromb Vasc Biol. (2012) 32(9):2045–51. 10.1161/ATVBAHA.108.179705 22895665 PMC3422754

[2] Boren J Chapman MJ Krauss RM Packard CJ Bentzon JF Binder CJ Low-Density lipoproteins cause atherosclerotic cardiovascular disease: pathophysiological, genetic, and therapeutic insights: a consensus statement from the European atherosclerosis society consensus panel. Eur Heart J. (2020) 41(24):2313–30. 10.1093/eurheartj/ehz962 32052833 PMC7308544

[3] Grundy SM Stone NJ Bailey AL Beam C Birtcher KK Blumenthal RS 2018 Aha/Acc/Aacvpr/Aapa/Abc/Acpm/Ada/Ags/Apha/Aspc/Nla/Pcna guideline on the management of blood cholesterol: a report of the American College of Cardiology/American Heart Association task force on clinical practice guidelines. Circulation. (2019) 139(25):e1082–e143. 10.1161/CIR.0000000000000625 30586774 PMC7403606

[4] Mach F Baigent C Catapano AL Koskinas KC Casula M Badimon L 2019 Esc/eas guidelines for the management of dyslipidaemias: lipid modification to reduce cardiovascular risk. Eur Heart J. (2020) 41(1):111–88. 10.1093/eurheartj/ehz455 31504418

[5] Cholesterol Treatment Trialists C, Baigent C Blackwell L Emberson J Holland LE Reith C Bhala N Efficacy and safety of more intensive lowering of ldl cholesterol: a meta-analysis of data from 170,000 participants in 26 randomised trials. Lancet (2010) 376(9753):1670–81. 10.1016/S0140-6736(10)61350-5 21067804 PMC2988224

[6] Sabatine MS Giugliano RP Keech AC Honarpour N Wiviott SD Murphy SA Evolocumab and clinical outcomes in patients with cardiovascular disease. N Engl J Med. (2017) 376(18):1713–22. 10.1056/NEJMoa1615664 28304224

[7] Hoogeveen RC Ballantyne CM. Residual cardiovascular risk at low ldl: remnants, lipoprotein(a), and inflammation. Clin Chem. (2021) 67(1):143–53. 10.1093/clinchem/hvaa252 33257928 PMC7793228

[8] Nordestgaard BG Varbo A. Triglycerides and cardiovascular disease. Lancet. (2014) 384(9943):626–35. 10.1016/S0140-6736(14)61177-6 25131982

[9] Varbo A Nordestgaard BG. Directly measured vs. Calculated remnant cholesterol identifies additional overlooked individuals in the general population at higher risk of myocardial infarction. Eur Heart J. (2021) 42(47):4833–43. 10.1093/eurheartj/ehab293 34023898

[10] Nakamura T Obata JE Hirano M Kitta Y Fujioka D Saito Y Predictive value of remnant lipoprotein for cardiovascular events in patients with coronary artery disease after achievement of ldl-cholesterol goals. Atherosclerosis. (2011) 218(1):163–7. 10.1016/j.atherosclerosis.2011.04.040 21605862

[11] Nguyen SV Nakamura T Kugiyama K. High remnant lipoprotein predicts recurrent cardiovascular events on statin treatment after acute coronary syndrome. Circ J. (2014) 78(10):2492–500. 10.1253/circj.cj-14-0380 25168189

[12] Castaner O Pinto X Subirana I Amor AJ Ros E Hernaez A Remnant cholesterol, not ldl cholesterol, is associated with incident cardiovascular disease. J Am Coll Cardiol. (2020) 76(23):2712–24. 10.1016/j.jacc.2020.10.008 33272365

[13] Quispe R Martin SS Michos ED Lamba I Blumenthal RS Saeed A Remnant cholesterol predicts cardiovascular disease beyond ldl and apob: a primary prevention study. Eur Heart J. (2021) 42(42):4324–32. 10.1093/eurheartj/ehab432 34293083 PMC8572557

[14] Baratta F Cocomello N Coronati M Ferro D Pastori D Angelico F Cholesterol remnants, triglyceride-rich lipoproteins and cardiovascular risk. Int J Mol Sci. (2023) 24(5):4268–79. 10.3390/ijms24054268 36901696 PMC10002331

[15] Gugliucci A. Triglyceride-Rich lipoprotein metabolism: key regulators of their flux. J Clin Med. (2023) 12(13):4399–423. 10.3390/jcm12134399 37445434 PMC10342861

[16] Chapman MJ Ginsberg HN Amarenco P Andreotti F Boren J Catapano AL Triglyceride-rich lipoproteins and high-density lipoprotein cholesterol in patients at high risk of cardiovascular disease: evidence and guidance for management. Eur Heart J. (2011) 32(11):1345–61. 10.1093/eurheartj/ehr112 21531743 PMC3105250

[17] Sandesara PB Virani SS Fazio S Shapiro MD. The forgotten lipids: triglycerides, remnant cholesterol, and atherosclerotic cardiovascular disease risk. Endocr Rev. (2019) 40(2):537–57. 10.1210/er.2018-00184 30312399 PMC6416708

[18] Miller YI Choi SH Fang L Tsimikas S. Lipoprotein modification and macrophage uptake: role of pathologic cholesterol transport in atherogenesis. Subcell Biochem. (2010) 51:229–51. 10.1007/978-90-481-8622-8_8 20213546

[19] Varbo A Benn M Tybjaerg-Hansen A Nordestgaard BG. Elevated remnant cholesterol causes both low-grade inflammation and ischemic heart disease, whereas elevated low-density lipoprotein cholesterol causes ischemic heart disease without inflammation. Circulation. (2013) 128(12):1298–309. 10.1161/CIRCULATIONAHA.113.003008 23926208

[20] Wang YI Bettaieb A Sun C DeVerse JS Radecke CE Mathew S Triglyceride-Rich lipoprotein modulates endothelial vascular cell adhesion molecule (vcam)-1 expression via differential regulation of endoplasmic Reticulum stress. PloS one. (2013) 8(10):e78322. 10.1371/journal.pone.0078322 24205197 PMC3804477

[21] Smolen JS Aletaha D McInnes IB. Rheumatoid arthritis. Lancet. (2016) 388(10055):2023–38. 10.1016/S0140-6736(16)30173-8 27156434

[22] Avina-Zubieta JA Thomas J Sadatsafavi M Lehman AJ Lacaille D. Risk of incident cardiovascular events in patients with rheumatoid arthritis: a meta-analysis of observational studies. Ann Rheum Dis. (2012) 71(9):1524–9. 10.1136/annrheumdis-2011-200726 22425941

[23] Choy E Ganeshalingam K Semb AG Szekanecz Z Nurmohamed M. Cardiovascular risk in rheumatoid arthritis: recent advances in the understanding of the pivotal role of inflammation, risk predictors and the impact of treatment. Rheumatology (Oxford). (2014) 53(12):2143–54. 10.1093/rheumatology/keu224 24907149 PMC4241890

[24] Im CH Kim NR Kang JW Kim JH Kang JY Bae GB Inflammatory burden interacts with conventional cardiovascular risk factors for carotid plaque formation in rheumatoid arthritis. Rheumatology (Oxford). (2015) 54(5):808–15. 10.1093/rheumatology/keu376 25305139

[25] Chen DY Chen YM Hsieh TY Hsieh CW Lin CC Lan JL. Significant effects of biologic therapy on lipid profiles and insulin resistance in patients with rheumatoid arthritis. Arthritis Res Ther. (2015) 17(1):52. 10.1186/s13075-015-0559-8 25889426 PMC4384305

[26] Myasoedova E Crowson CS Kremers HM Roger VL Fitz-Gibbon PD Therneau TM Lipid paradox in rheumatoid arthritis: the impact of Serum lipid measures and systemic inflammation on the risk of cardiovascular disease. Ann Rheum Dis. (2011) 70(3):482–7. 10.1136/ard.2010.135871 21216812 PMC3058921

[27] Kremer JM Genovese MC Keystone E Taylor PC Zuckerman SH Ruotolo G Effects of baricitinib on lipid, apolipoprotein, and lipoprotein particle profiles in a phase iib study of patients with active rheumatoid arthritis. Arthritis Rheumatol. (2017) 69(5):943–52. 10.1002/art.40036 28029752

[28] Charles-Schoeman C Gonzalez-Gay MA Kaplan I Boy M Geier J Luo Z Effects of tofacitinib and other dmards on lipid profiles in rheumatoid arthritis: implications for the rheumatologist. Semin Arthritis Rheum. (2016) 46(1):71–80. 10.1016/j.semarthrit.2016.03.004 27079757

[29] Aletaha D Neogi T Silman AJ Funovits J Felson DT Bingham CO 3rd, 2010 Rheumatoid arthritis classification criteria: an American college of rheumatology/European league against rheumatism collaborative initiative. Ann Rheum Dis (2010) 69(9):1580–8. 10.1136/ard.2010.138461 20699241

[30] Prevoo ML van ‘t Hof MA Kuper HH van Leeuwen MA van de Putte LB van Riel PL. Modified disease activity scores that include twenty-eight-joint counts. Development and validation in a prospective longitudinal study of patients with rheumatoid arthritis. Arthritis Rheum. (1995) 38(1):44–8. 10.1002/art.1780380107 7818570

[31] Ledingham J Deighton C, British Society for Rheumatology Standards G, Audit Working G. Update on the British Society for Rheumatology guidelines for prescribing tnfalpha blockers in adults with rheumatoid arthritis (update of previous guidelines of April 2001). Rheumatology (Oxford) (2005) 44(2):157–63. 10.1093/rheumatology/keh464 15637039

[32] Nordestgaard BG Langsted A Mora S Kolovou G Baum H Bruckert E Fasting is not routinely required for determination of a lipid profile: clinical and laboratory implications including flagging at desirable concentration cut-points-a joint consensus statement from the European Atherosclerosis Society and European Federation of Clinical Chemistry and Laboratory Medicine. Eur Heart J. (2016) 37(25):1944–58. 10.1093/eurheartj/ehw152 27122601 PMC4929379

[33] Xiang QY Tian F Lin QZ Du X Zhang SL Gui YJ Comparison of remnant cholesterol levels estimated by calculated and measured ldl-C levels in Chinese patients with coronary heart disease. Clin Chim Acta. (2020) 500:75–80. 10.1016/j.cca.2019.09.020 31655058

[34] Mishra BH Mishra PP Mononen N Hilvo M Sievanen H Juonala M Lipidomic architecture shared by subclinical markers of osteoporosis and atherosclerosis: the cardiovascular risk in young Finns study. Bone. (2020) 131:115160. 10.1016/j.bone.2019.115160 31759205

[35] Chang CK Chang KH Cheng WC Chen PK Chiang EI Chang SH Lipid metabolomic signature might predict subclinical atherosclerosis in patients with active rheumatoid arthritis. Clin Exp Rheumatol. (2023) 41(5):1120–8. 10.55563/clinexprheumatol/int08c 36200949

[36] Charles-Schoeman C Buch MH Dougados M Bhatt DL Giles JT Ytterberg SR Risk of Major adverse cardiovascular events with tofacitinib versus tumour necrosis factor inhibitors in patients with rheumatoid arthritis with or without a history of atherosclerotic cardiovascular disease: a *post hoc* analysis from oral surveillance. Ann Rheum Dis. (2023) 82(1):119–29. 10.1136/ard-2022-222259 36137735 PMC9811099

[37] Varbo A Benn M Tybjaerg-Hansen A Jorgensen AB Frikke-Schmidt R Nordestgaard BG. Remnant cholesterol as a causal risk factor for ischemic heart disease. J Am Coll Cardiol. (2013) 61(4):427–36. 10.1016/j.jacc.2012.08.1026 23265341

[38] Dessein PH Joffe BI. Insulin resistance and impaired Beta cell function in rheumatoid arthritis. Arthritis Rheum. (2006) 54(9):2765–75. 10.1002/art.22053 16947779

[39] Yan Y La R Jiang M Xu W Jiang D Wang S The association between remnant cholesterol and rheumatoid arthritis: insights from a large population study. Lipids Health Dis. (2024) 23(1):38. 10.1186/s12944-024-02033-z 38326904 PMC10848346

[40] Huh JH Han KD Cho YK Roh E Kang JG Lee SJ Remnant cholesterol and the risk of cardiovascular disease in type 2 diabetes: a nationwide longitudinal cohort study. Cardiovasc Diabetol. (2022) 21(1):228. 10.1186/s12933-022-01667-6 36324177 PMC9632127

[41] Lindhardsen J Ahlehoff O Gislason GH Madsen OR Olesen JB Torp-Pedersen C The risk of myocardial infarction in rheumatoid arthritis and diabetes Mellitus: a danish nationwide cohort study. Ann Rheum Dis. (2011) 70(6):929–34. 10.1136/ard.2010.143396 21389043

[42] Varbo A Nordestgaard BG. Remnant cholesterol and triglyceride-rich lipoproteins in atherosclerosis progression and cardiovascular disease. Arterioscler Thromb Vasc Biol. (2016) 36(11):2133–5. 10.1161/ATVBAHA.116.308305 27784698

[43] Zou YW Wu T Li QH Ma JD Pan J Lu Y Association of Serum concentrations of remnant cholesterol with incident cardiovascular disease in patients with rheumatoid arthritis: a real-world data from 2001 to 2022. Int J Cardiol. (2024) 405:131947. 10.1016/j.ijcard.2024.131947 38458390

[44] Colaco K Lee KA Akhtari S Winer R Welsh P Sattar N Targeted metabolomic profiling and prediction of cardiovascular events: a prospective study of patients with psoriatic arthritis and psoriasis. Ann Rheum Dis. (2021) 80(11):1429–35. 10.1136/annrheumdis-2021-220168 34049856

[45] Smolen JS Breedveld FC Burmester GR Bykerk V Dougados M Emery P Treating rheumatoid arthritis to target: 2014 update of the recommendations of an international task force. Ann Rheum Dis. (2016) 75(1):3–15. 10.1136/annrheumdis-2015-207524 25969430 PMC4717393

[46] Kerschbaumer A Sepriano A Bergstra SA Smolen JS van der Heijde D Caporali R Efficacy of synthetic and biological dmards: a systematic literature review informing the 2022 update of the eular recommendations for the management of rheumatoid arthritis. Ann Rheum Dis. (2023) 82(1):95–106. 10.1136/ard-2022-223365 36368906

[47] Roubille C Richer V Starnino T McCourt C McFarlane A Fleming P The effects of tumour necrosis factor inhibitors, methotrexate, non-steroidal anti-inflammatory drugs and corticosteroids on cardiovascular events in rheumatoid arthritis, psoriasis and psoriatic arthritis: a systematic review and meta-analysis. Ann Rheum Dis. (2015) 74(3):480–9. 10.1136/annrheumdis-2014-206624 25561362 PMC4345910

[48] Kastrati K Aletaha D Burmester GR Chwala E Dejaco C Dougados M A systematic literature review informing the consensus statement on efficacy and safety of pharmacological treatment with interleukin-6 pathway inhibition with biological dmards in immune-mediated inflammatory diseases. RMD Open. (2022) 8(2):e002359–81. 10.1136/rmdopen-2022-002359 36260501 PMC9462104

[49] Souto A Salgado E Maneiro JR Mera A Carmona L Gomez-Reino JJ. Lipid profile changes in patients with chronic inflammatory arthritis treated with biologic agents and tofacitinib in randomized clinical trials: a systematic review and meta-analysis. Arthritis Rheumatol. (2015) 67(1):117–27. 10.1002/art.38894 25303044

[50] Makris A Barkas F Sfikakis PP Liberopoulos E Agouridis AP. The effect of upadacitinib on lipid profile and cardiovascular events: a meta-analysis of randomized controlled trials. J Clin Med. (2022) 11(23):6984. 10.3390/jcm11236894 36498468 PMC9740350

[51] Le NA Innis-Whitehouse W Li X Bakker-Arkema R Black D Brown WV. Lipid and apolipoprotein levels and distribution in patients with hypertriglyceridemia: effect of triglyceride reductions with atorvastatin. Metabolism: Clinical and Experimental. (2000) 49(2):167–77. 10.1016/s0026-0495(00)91169-7 10690940

[52] Naerr GW Rein P Saely CH Drexel H. Effects of synthetic and biological disease modifying antirheumatic drugs on lipid and lipoprotein parameters in patients with rheumatoid arthritis. Vascul Pharmacol. (2016) 81:22–30. 10.1016/j.vph.2016.01.006 26903239

[53] Yousri NA Bayoumy K Elhaq WG Mohney RP Emadi SA Hammoudeh M Large scale metabolic profiling identifies novel steroids linked to rheumatoid arthritis. Sci Rep. (2017) 7(1):9137. 10.1038/s41598-017-05439-1 28831053 PMC5567269

