# Supplementary material for: Elevated remnant cholesterol as a potential predictor for cardiovascular events in rheumatoid arthritis patients
Source: Front Cardiovasc Med. 2024 Sep 9;11:1449219. doi: 10.3389/fcvm.2024.1449219 (PMC11423425; doi:10.3389/fcvm.2024.1449219)
Supplement: Supplementary file 1 [file Table1.docx]

**Supplementary Table 1.** Demographic data and laboratory findings in rheumatoid arthritis (RA) patients and healthy control participants#

|  | RA (n=114) | Healthy control (n=41) |
| --- | --- | --- |
| Age at entry, years | 59.4±12.0 | 55.7±10.2 |
| Female proportion, n (%) | 88 (77.2%) | 29 (70.7%) |
| Body mass index, kg/m2 | 23.8±3.9 | 22.8±2.2 |
| Total cholesterol, mg/dL | 196.3±43.5 | 194.1±42.1 |
| Triglyceride, mg/dL | 107.2±71.4 | 91.4±37.6 |
| LDL-C, mg/dL | 112.8±34.4 | 115.4±36.9 |
| HDL-C, mg/dL | 61.4±15.2 | 62.7±14.1 |
| Atherogenic index | 3.35±0.99 | 3.22±0.79 |
| RC, mg/dL | 22.1±12.1 | 16.0±11.1 |
| Hypertension, n (%) | 29 (25.4%) | 2 (9.5%) |
| Diabetes mellitus, n (%) | 6 (5.3%) | 0 (0%) |
| Current smoker, n (%) | 16 (14.0%) | 3 (14.3%) |

#Data are presented as mean ± SD, or number (percentage).

**Supplementary Table 2.** Demographic data and laboratory findings in RA patients with or without cardiovascular event (CVE) #

|  | RA with CVE  (n=19) | RA without CVE  (n=95) |
| --- | --- | --- |
| Age at entry, years | 66.5±13.3 | 58.0±11.2 |
| Female proportion, n (%) | 12 (63.2%) | 76 (80.0%) |
| Disease duration, years | 7.7±2.7 | 6.6±2.1 |
| Body mass index, kg/m^2^ | 24.4±3.8 | 23.7±3.9 |
| RF positivity, n (%) | 12 (63.2%) | 70 (73.7%) |
| ACPA positivity, n (%) | 12 (63.2%) | 72 (76.6%) |
| Baseline ESR, mm/1^st^ hr | 28.3±20.3 | 29.9±20.0 |
| Baseline CRP, mg/dL | 1.65±3.82 | 2.06±3.33 |
| DAS28-ESR at baseline | 6.15±1.03 | 5.65±1.14 |
| Total cholesterol, mg/dL | 226.9±48.0 | 190.2±40.0 |
| Triglyceride, mg/dL | 179.5±111.9 | 92.6±49.3 |
| LDL-C, mg/dL | 130.4±37.3 | 109.3±32.9 |
| HDL-C, mg/dL | 58.1±14.1 | 62.1±15.4 |
| Atherogenic index | 4.08±1.23 | 3.20±0.87 |
| RC, mg/dL | 38.4±15.9 | 18.8±7.9 |
| Daily corticosteroids, mg/day | 3.6±3.7 | 4.9±3.8 |
| Baseline used csDMARDs |  |  |
| Methotrexate, n (%) | 16 (84.2%) | 76 (80.0%) |
| Sulfasalazine, n (%) | 13 (68.4%) | 67 (70.5%) |
| Hydroxychloroquine, n (%) | 12 (63.2%) | 58 (61.1%) |
| bDMARDs/JAKi after lipid investigation |  |  |
| TNF-α inhibitors, n (%) | 3 (15.8%) | 18 (18.9%) |
| Non-TNF-α inhibitors, n (%) | 7 (36.8%) | 29 (30.5%) |
| JAK inhibitors, n (%) | 9 (47.4%) | 48 (50.5%) |
| The use of statin, n (%) | 10 (52.6%) | 20 (21.1%) |
| Hypertension, n (%) | 9 (47.4%) | 20 (21.1%) |
| Diabetes mellitus, n (%) | 2 (10.5%) | 4 (4.2%) |
| Current smoker, n (%) | 1 (5.3%) | 15 (15.8%) |

#Data are presented as mean ± SD, or number (percentage). RA: rheumatoid arthritis; RF: rheumatoid factor; ACPA: anti-citrullinated peptide antibodies; ESR: erythrocyte sedimentation rate; CRP: C-reactive protein; DAS28: disease activity score for 28-joints; LDL: low-density lipoprotein; HDL: high-density lipoprotein; RC: remnant cholesterol; csDMARDs: conventional synthetic disease-modifying anti-rheumatic drugs; bDMARDs: biologic DMARDs; JAKi: Janus kinase inhibitors; TNF: tumor necrosis factor.

**Supplementary Table 3.** Univariate and multivariate regression analysis of baseline lipid profile and RC for predicting the incident cardiovascular events in 114 patients with RA

| Risk factors (**univariate**) | Odds ratio | 95% confidence interval | *p*-value |
| --- | --- | --- | --- |
| **Age** | **1.077** | **1.022 – 1.135** | **0.006** |
| Gender (Female) | 0.429 | 0.149 – 1.236 | 0.117 |
| **Total cholesterol** | **1.020** | **1.008 – 1.033** | **0.002** |
| **Triglyceride** | **1.017** | **1.007 – 1.027** | **0.001** |
| **LDL-C** | **1.018** | **1.003 – 1.033** | **0.018** |
| HDL-C | 0.982 | 0.949 – 1.016 | 0.299 |
| **Atherogenic index** | **2.225** | **1.361 – 3.636** | **0.001** |
| CRP | 0.959 | 0.807 – 1.140 | 0.634 |
| JAKi treatment | 0.789 | 0.239 – 2.609 | 0.698 |
| Non-TNFi treatment | 0.855 | 0.281 – 2.599 | 0.782 |
| TNFi treatment | 0.627 | 0.131 – 3.002 | 0.560 |
| **RC** | **1.198** | **1.110 – 1.293** | **<0.001** |
| Risk factors (**multivariate**) | Odds ratio | 95% confidence interval | *p*-value |
| **Age** | **1.242** | **1.062 – 1.453** | **0.007** |
| Gender (Female) | 0.102 | 0.007 – 1.463 | 0.093 |
| LDL-C | 1.300 | 0.914 – 1.847 | 0.144 |
| Total cholesterol | 0.812 | 0.622 – 1.061 | 0.127 |
| Triglyceride | 1.001 | 0.979 – 1.024 | 0.917 |
| Atherogenic index | 0.021 | 0.000 – 7.191 | 0.195 |
| CRP | 1.034 | 0.779 – 1.373 | 0.815 |
| JAKi treatment | 0.296 | 0.011 – 7.844 | 0.466 |
| Non-TNFi treatment | 0.326 | 0.021 – 4.958 | 0.420 |
| TNFi treatment | 0.831 | 0.030 – 23.320 | 0.913 |
| **RC** | **1.997** | **1.131 – 3.526** | **0.017** |

The incident cardiovascular events include fatal or non-fatal MI, stable or unstable angina pectoris, ischemic stroke, and reversible focal neurological defects with imaging evidence of a new cerebral lesion compatible with ischemia. RA: rheumatoid arthritis; HDL-C: high-density lipoprotein cholesterol; LDL-C: low-density lipoprotein cholesterol; RC: remnant cholesterol; Atherogenic index corresponds to the ratio of total cholesterol/HDL-C. Bold values indicate statistically significant results.
